# Layered Zirconium Phosphate Nanocarriers for Mitoxantrone: Advancing Targeted Chemotherapy

**DOI:** 10.1021/acsomega.6c01791

**Published:** 2026-06-12

**Authors:** Aleannette López-Cubero, María M. Sánchez, Getsemary Cabrera-Rivera, Armando Santiago, Yairisis Rivera, Sehwan Jang, Millie L. González, Magaly Martínez-Ferrer, Jorge L. Colón

**Affiliations:** † Department of Chemistry, 19878 University of Puerto Rico, 17 Ave. Universidad STE 1701, San Juan 00925-2537, Puerto Rico; ‡ Division of Cancer Clinical & Translational Research, Comprehensive Cancer Center, University of Puerto Rico, San Juan 00921, Puerto Rico; § Deanship of Research, Medical Sciences Campus, 12320 University of Puerto Rico, San Juan 00921, Puerto Rico; ∥ Department of Pharmaceutical Sciences, Medical Sciences Campus, University of Puerto Rico, San Juan 00921, Puerto Rico

## Abstract

Layered zirconium phosphate nanoparticles (ZrPs), inorganic structures with a layered composition of zirconium atoms linked by phosphate groups, exhibit no cytotoxic effects on healthy or cancer cells, indicating a strong safety profile. We are investigating ZrP as a carrier for mitoxantrone (MTX), an anticancer drug that inhibits topoisomerase II and disrupts DNA synthesis but lacks selectivity between healthy and cancer cells. MTX shows prolonged retention in the body, with potential systemic toxicity affecting thyroid, hepatic, and cardiac tissues. We report a strategy to reduce systemic toxicity and protect healthy tissues from MTX by intercalating MTX into ZrP (MTX@ZrP), determining the conditions that affect MTX’s release, and evaluating the cytotoxicity of MTX@ZrP on PC3 prostate cancer cells. MTX was intercalated within ZrP, forming MTX@ZrP nanoparticles, which were then characterized by Fourier transform infrared spectroscopy, scanning electron microscopy/energy-dispersive X-ray spectroscopy, z-potential analysis, and X-ray powder diffraction (XRPD) at different MTX:ZrP molar ratios. XRPD showed that MTX@ZrP has an expanded interlayer distance of 20.0 Å compared to the 7.6 Å interlayer distance of α-ZrP. Thermogravimetric analysis performed on all molar ratios of MTX to ZrP showed that the 1:1 MTX@ZrP molar ratio material had the highest drug loading of 13.8%. A drug release study to determine conditions affecting MTX liberation from ZrP at different pH levels using simulated body fluid and artificial lysosomal fluid showed that this process is pH-dependent. Cell viability studies with the androgen receptor-negative prostate cancer cell line PC3 showed that MTX@ZrP produces a cytotoxic effect. These findings help us envision a possible drug delivery approach that may greatly minimize adverse effects and harm to healthy tissues, offering promises for a more bearable cancer treatment.

## Graphical abstract

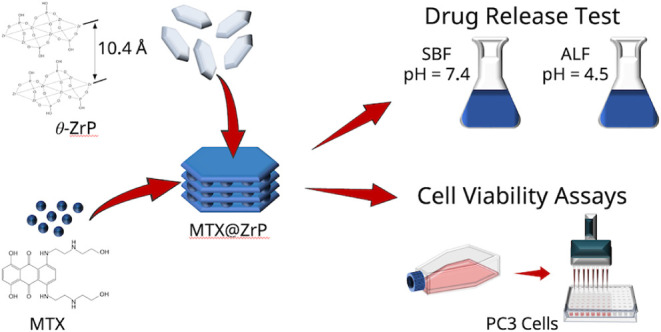

## 1 Introduction

Conventional chemotherapy often lacks selectivity between cancerous and healthy cells, resulting in significant damage and severe side effects like vomiting, diarrhea, and fatigue, which can jeopardize patient safety.^1,2^ To improve targeting and minimize harm, nanoparticles have been proposed as drug carriers to enhance drug stability, help drugs evade the immune system, target specific cells, efficiently enter cancer cells, decrease toxic side effects, and improve pharmacokinetic and pharmacodynamic drug properties.^3–8^ Various types of nanoparticles have been developed, including liposomes,^9^ polymeric micelles,^10,11^ lipids,^12^ and inorganic layered nanomaterials (ILN).^13–20^ However, many of these nanoparticles face challenges such as poor oral bioavailability, instability in serum conditions, and potential toxicity.^3^ In this study, we investigated the use of the layered inorganic material zirconium phosphate (ZrP) as a potential anticancer drug nanocarrier.

ZrP is an inorganic layered material composed of zirconium atoms, aligned in a plane and covalently bonded to six oxygen atoms belonging to different adjacent tetrahedral phosphate groups forming octahedral ZrO_6_ units (Figure 1).^21,22^ The fourth oxygen of the phosphate group is attached to a proton pointing toward the interlaminar region,^22^ allowing the intercalation of anticancer drugs by ion exchange with the protons of the phosphate groups^23^ or acid–base interactions between a protonated Brønsted base and the deprotonated oxygen of the phosphate.^22^ In addition, two-dimensional ^31^P NMR experiments have shown that organic intercalant species can be stabilized within ZrP due to strong hydrogen-bond interactions.^24^

**Figure 1:**
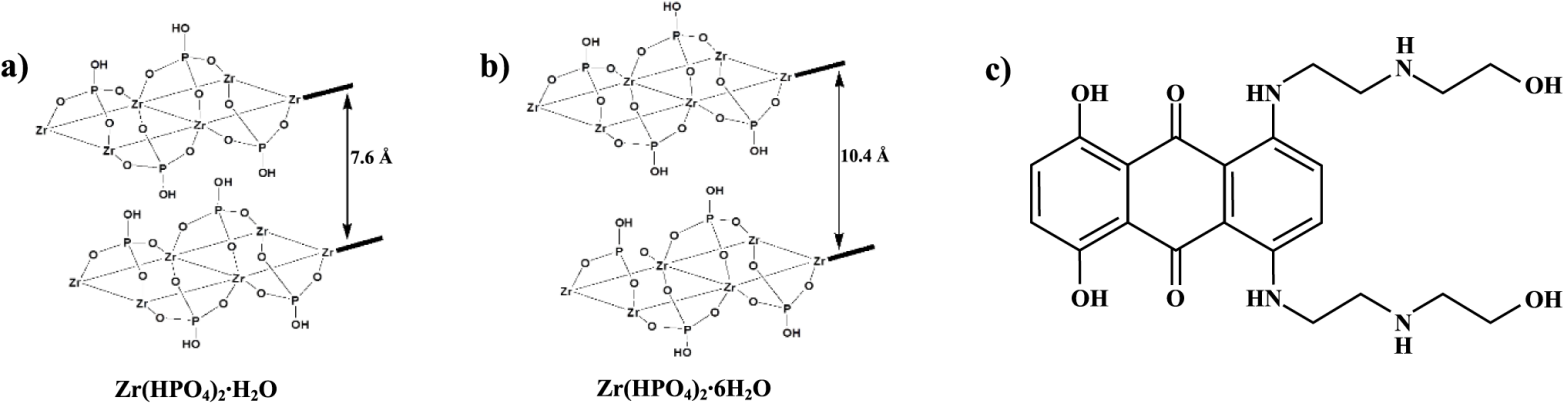
Schematic representation of the structures of a) α-ZrP, b) θ-ZrP, and c) MTX.

ZrP has been used as a drug delivery agent.^25–28^ The α-phase of ZrP (Zr­(HPO_4_)_2_·H_2_O, α-ZrP) has an interlayer distance of just 7.6 Å, limiting the size of species that can be directly intercalated.^23,29^ In contrast, the highly hydrated phase of ZrP (Zr­(HPO_4_)_2_·6H_2_O, θ-ZrP) has an interlaminar distance of 10.4 Å, significantly increasing its capacity to intercalate larger cations over that of α-ZrP. θ-ZrP has been used to successfully intercalate several bioactive compounds^23,24^ such as cisplatin,^30^ molybdenum dichloride,^31^ doxorubicin,^13,23^ and insulin.^32^

ZrP nanoparticles are promising candidates for use as anticancer drug carriers since they can be modified for targeted delivery, have a high loading capacity, and remain stable under biological conditions.^21,30,32–34^ Cell studies demonstrate that ZrP is noncytotoxic to various normal and cancer cell lines, including human kidney, breast, ovarian, and macrophage cells.^21,23^ In addition, the nanoplatelet shape of ZrP also enhances its interaction with endothelial tissue, promoting tumbling, which aids in extravasation and improves the enhanced permeability and retention (EPR) effect.^35–39^

The EPR effect is a passive targeting mechanism in macromolecule- and nanoparticle-based cancer therapy that enables the preferential and progressive accumulation of therapeutic agents in tumor tissues.^36,40–45^ Unlike normal vasculature, tumor blood vessels exhibit structural abnormalities, which depend on the tumor type, stage, and location, including extensive endothelial gaps (fenestrations) ranging from 100 to 600 nm. The blood vessel fenestrations facilitate the extravasation of nanoparticles into the tumor microenvironment.^1,36,46,47^ Moreover, impaired lymphatic drainage prevents efficient clearance, ensuring prolonged nanoparticle retention and maximized cellular uptake.^36,48^ This combination of vascular leakage and impaired lymphatic function significantly enhances drug delivery, making the EPR effect a cornerstone of nanoparticle-mediated cancer treatment. However, there are still challenges related to passive targeting; there is only a 0.7% median delivery efficiency of nanoparticles administered to a solid tumor.^45,49^ Strategies to overcome these challenges are being developed.^50–56^

Here, we report the successful intercalation of the anticancer drug mitoxantrone (MTX) into ZrP nanoparticles, referred to as MTX@ZrP. MTX is an anthracenedione that inhibits the nuclear enzyme topoisomerase II, leading to DNA breaks and delayed cell-cycle progression.^57–60^ While MTX is effective against various types of cancer such as leukemia, non-Hodgkin’s lymphoma, breast cancer, and prostate cancer, its administration can lead to several side effects.^57^ These may include nausea, diarrhea, leukopenia, neutropenia, thrombocytopenia, anemia, and increased cardiotoxicity in patients.^57,59,61,62^ Strategies to minimize MTX side effects include using nanocarriers for drug delivery to reduce the systemic toxicity of the drug and achieve higher tumor specificity.^62–64^

Since these side effects could be minimized by using ZrP as a drug carrier, we report the release profile of MTX from ZrP nanoparticles in the buffers simulated body fluid (SBF, pH = 7.4) and artificial lysosomal fluid (ALF, pH = 4.5), and MTX@ZrP’s effect in the cell viability of human androgen receptor-negative (PC3) prostate cancer cells.

## 2 Reactants and Methods

### 2.1 Reactants

Zirconyl chloride octahydrate (ZrOCl_2_·8H_2_O, 98%) was obtained from Sigma-Aldrich and used without further purification. Phosphoric acid (H_3_PO_4_, 85% v/v) (ACS Reagent) was obtained from Fisher Chemical, and mitoxantrone dichloride (97%) was obtained from AK Scientific. Nanopure water was obtained using a Gemini high-purity water system, model GMS-105 (18.2 MΩ).

### 2.2 Synthesis of θ-ZrP

θ-ZrP was synthesized based on the method reported by Marti et al.^65^ A 200 mL volume of 6 M H_3_PO_4_ was preheated in an oil bath at 94 °C in a 500 mL round-bottom flask, followed by the dropwise addition of 200 mL of 0.05 M ZrOCl_2_·8H_2_O. The solution was refluxed with constant stirring at 94 °C for 48 h. After refluxing, the product was centrifuged to remove the supernatant and washed several times with water, obtaining an aqueous suspension.

### 2.3 Intercalation of MTX into θ-ZrP

An aqueous solution of MTX was added to an aqueous suspension of θ-ZrP at different ZrP:MTX molar ratios (50:1, 20:1, 10:1, 5:1, and 1:1). The suspensions were stirred continuously on an orbital shaker for 5 days at room temperature. After this period, the suspensions were filtered using 0.22 μm PVDF filters (Durapore) under vacuum. The solid residue was washed thoroughly with water, dried in a vacuum dryer for 1 day, and pulverized for characterization and studies.

### 2.4 Characterization of MTX@ZrP

#### 2.4.1 X-ray Powder Diffraction (XRPD)

XRPD analyses were performed from 2° to 40° (2θ angle) using a Rigaku SuperNova single-source microfocus HyPix3000 diffractometer, with Cu Kα radiation at 300 K (1.5406 Å). Data reduction was performed using the program CrysAlisPro (CrysAlis PRO 1.171.39.46, Rigaku OD, 2018). The interlayer distances were determined using Bragg’s Law (*n*λ = 2*d*_*hkl*_ sinθ, where λ is the wavelength of the X-ray source, *d*_*hkl*_ is the interlayer distance between planes in the unit cell, and θ is the diffraction angle).

#### 2.4.2 Fourier Transform Infrared Spectroscopy (FTIR)

The FTIR measurements were performed from 400 cm^–1^ to 4000 cm^–1^ using a Bruker Tensor-27 with a Helios attenuated total reflectance (ATR) accessory and a single-bounce diamond crystal, with the OPUS Data Collection Program for the analysis.

#### 2.4.3 Scanning Electron Microscopy/Energy-Dispersive X-ray Spectroscopy (SEM/EDS)

MTX@ZrP samples were sputter-coated with a thin gold film (∼30 s) using a PELCO SC-7 Auto Sputter Coater before SEM images. SEM/EDS images and spectra were obtained using a JEOL 6480 LV scanning electron microscope with an EDAX X-ray fluorescence detecting unit with a Genesis 2000 detector.

#### 2.4.4 Z-Potential

The electrostatic potential of ZrP nanoparticles’ surface was determined using z-potential analysis with a Malvern Zetasizer Nano-ZS, equipped with a He–Ne laser (632.8 nm). The samples were prepared in deionized water.

#### 2.4.5 Thermogravimetric Analysis (TGA)

The amount of MTX loaded into ZrP nanoparticles was determined by TGA using a TGA Q500 TA Instrument (New Castle, DE). The temperature was ramped from 30 to 700 °C at 5 °C min^–1^ under a flow of N_2_ up to 800 °C.

### 2.5 In Vitro Drug Release Tests (DRTs)

#### 2.5.1 Experimental Conditions

The DRTs used the MTX@ZrP with the highest amount of intercalated MTX, as determined by TGA. The first DRT of MTX@ZrP was performed in ALF, which mimics the lysosomal environment at pH 4.5 based on the method reported by Stopford et al.^66^ In a polypropylene tube, a 10 mL suspension of MTX@ZrP in ALF at a concentration of 0.03% w/v was placed and incubated in a thermomixer (Eppendorf AG, Germany) at 37 °C and 400 rpm. At specific time intervals, several aliquots were taken, and the volume extracted from the MTX@ZrP suspension was replaced with fresh ALF. Each aliquot was filtered using a Chromafil disposable syringe filter (PET-20/15 MS) and analyzed with a Cary 100 Series UV–Vis spectrophotometer (Agilent Technologies) in the wavelength range of 200–800 nm via UV–Vis spectrophotometry. Measurements were conducted at room temperature using UV Cary Scan software (version 20.0.470), and absorbances at 662 nm were used to determine the concentration of each aliquot as well as the cumulative percentage of MTX release. The study was performed in triplicate.

The second DRT was performed using these experimental conditions, but for MTX@ZrP in SBF, which was prepared following the protocol described by Kokubo and Takadama in 2006 and with a pH = 7.4.^67^

#### 2.5.2 Calibration Curves

Standard solutions of MTX were prepared from a stock solution of known concentration through a series of consecutive dilutions in volumetric flasks. The solvent used for these dilutions was either ALF or SBF, depending on the specific DRT performed. By utilizing the same standard solution for each concentration, two calibration curves were created. These curves were used to quantify the amount of MTX retained by the filter after filtration and to determine the actual concentration of each aliquot.

The first calibration curve was generated from filtered samples. Using its trendline equation, which was fitted with a second-degree polynomial, the MTX concentration in each filtered aliquot collected during the experiment was determined. The second calibration curve was obtained from unfiltered samples. The absorbance values were used to determine the percentage of MTX retained by the filter, according to the following formula:^68^

$$MTX\%\;\mathrm{Retention}=\left(1-\frac{\mathrm{Abs filtered}}{\mathrm{Abs unfiltered}}\right)\times 100$$

where Abs filtered and Abs unfiltered correspond to the absorbance values of filtered and unfiltered samples, respectively. Both calibration curves were analyzed using the same parameters and UV–vis spectrophotometer described in Section 2.5.1. A graph of MTX retention percentage versus concentration was plotted. Using the linear trendline equation, the actual concentration of MTX in each aliquot was determined.

#### 2.5.3 Determination of Cumulative Drug Release Percentage

The mass of the drug released in each aliquot was calculated using the following formula:

$$M_{a,n}=V_{a}\times C_{n}$$

where *M*_a,*n*_ is the mass of the aliquot at time *n*, *V*_a_ is the volume of the aliquot, and *C_n_* is the concentration of the aliquot at time *n*. The cumulative release percentage of the drug was calculated using the following formula:

$$\mathrm{Drug Cumulative Release}\%=\frac{V_{t}\times C_{n}+\overset{n-1}{\underset{i=1}{\sum }}\left(C_{i}\times V_{a}\right)}{M_{t}}\times 100$$

where *V*_t_ is the total volume of the drug suspension, *C_n_* is the concentration of the drug suspension at time *n*, $\overset{n-1}{\underset{i=1}{\sum }}\left(C_{i}\times V_{a}\right)$ is the summation of the total mass of each aliquot retrieved from the drug suspension, and *M*_t_ is the mass of the drug present in the suspension at time 0.

#### 2.5.4 Statistical Analysis

Statistical analysis was conducted to determine whether there were significant differences between each repetition of the study. This involved using the nonparametric Friedman test and, when necessary, post hoc Wilcoxon signed-rank tests with Bonferroni correction. Additionally, an analysis of variance for repeated measures (ANOVA) and Shapiro–Wilk normality tests were performed using the Statsmodels and SciPy in Python. The cumulative release profiles, calculated as the average from three independent repetitions, were fitted using nonlinear regression to evaluate the release kinetics. Four classical models—zero-order, first-order, Higuchi, and Korsmeyer–Peppas—were applied using SciPy’s curve fit function. To account for mixed mechanisms, a custom hybrid model was developed, defined as a weighted combination of the first-order and Korsmeyer–Peppas equations. The quality of the fits was assessed by comparing the coefficients of determination (*R* ^2^).

### 2.6 In Vitro Cell Viability Assays

#### 2.6.1 Cell Culture

PC3 cells were purchased from the American Type Culture Collection (ATCC) (Manassas, VA, USA), cultured in RPMI-1640 medium with l-glutamine (Gibco, NY, USA), and supplemented with 10% fetal bovine serum (FBS). Cells were maintained in a 5% CO_2_ humidified atmosphere at 37 °C.

#### 2.6.2 Cell Viability Assays

Cell viability was assessed using the MTS assay kit (AB 197010 from Abcam, USA) according to the manufacturer’s instructions. PC3 cells were cultured in a 96-well plate at a density of 10,000 cells per well. After 24 h of incubation, the cells were treated with ZrP, MTX@ZrP, and free MTX at concentrations equivalent to 0.75, 1.5, 5, 10, 15, and 20 μM MTX in fresh complete medium. The MTS reagent was added after 24, 48, and 72 h of treatment, followed by a 2-h incubation in a CO_2_ incubator. Absorbance was measured at 490 nm using an xMark microplate absorbance spectrophotometer (BIORAD, USA) and Microplate Manager software. The experiment was performed in triplicate, and the IC_50_ was determined by nonlinear regression fitting curve with GraphPad Prism software.

## 3 Results and Discussion

### 3.1 MTX@ZrP Characterization

Figure 2 shows the XRPD patterns of α-ZrP and MTX@ZrP at different MTX:ZrP molar ratios, which was prepared using θ-ZrP. The diffractogram of α-ZrP shows a prominent peak at 2θ = 11.6°, indicating the expected interlayer distance of 7.6 Å. This peak serves as a reference point for assessing the success of intercalation since the intercalated samples also undergo a drying process before obtaining their XRPD patterns, so unintercalated θ-ZrP converts to α-ZrP upon dehydration. If no diffraction peak appears at distances larger than 7.6 Å after the intercalation reaction process, that would indicate that the intercalation reaction was not successful.

**Figure 2:**
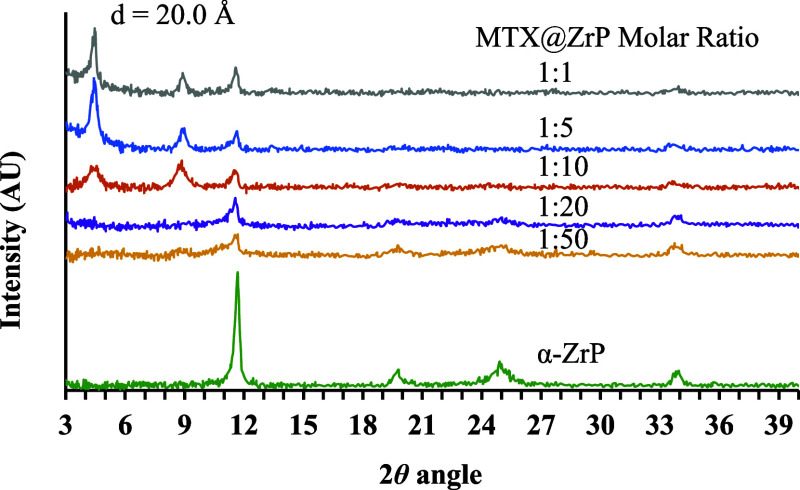
XRD patterns of α-ZrP and MTX@ZrP at different MTX:ZrP molar ratios.

The MTX@ZrP diffractograms show two additional peaks beyond those found in the α-ZrP diffractogram, starting at a 1:10 molar ratio of MTX to ZrP and increasing in intensity with higher concentrations of MTX during intercalation. These peaks show at 2θ = 4.4° and a second-order at 2θ = 8.89°, corresponding to an interlaminar distance of 20.0 Å. This interlayer distance is greater than that of α-ZrP, indicating successful intercalation. Since the thickness of a ZrP layer is 6.6 Å,^69,70^ the MTX contribution to the MTX@ZrP interlayer distance is 13.4 Å, consistent with one of the molecular dimensions of the MTX of 13.5 Å.^64^ Additionally, peaks at 2θ = 33.81° and 34.21° correspond to the 020 and 312̅ planes of ZrP, indicating that the α-type layers remain intact. The peak at 2θ = 11.6° implies that a mixed ZrP phase was produced due to the remaining presence of α-ZrP at those MTX loading levels.

FTIR spectroscopy measurements were performed to corroborate the successful intercalation of MTX within ZrP. Figure 3 shows the FTIR spectra of α-ZrP and MTX@ZrP at different molar ratios. The spectrum of α-ZrP shows three characteristic peaks from the vibrational modes of lattice water at 3590 cm^–1^, 3510 cm^–1^, and 1616 cm^–1^, and a broad band from hydrogen bonding at 3141 cm^–1^.^71^ Another peak at 1055 cm^–1^, corresponds to the P–O–H group stretching mode (Figure 3).^72^ On the other hand, the MTX IR spectrum shows peaks at 1570 cm^–1^ and 1615 cm^–1^, due to the C=O stretching of the quinone; at 1512 cm^–1^, due to C–N–H stretching of the quinone; at 1445 cm^–1^, due to the C–H bending; and at 1210 cm^–1^, due to the C–O stretching vibrational mode.^73^ The FTIR spectra of MTX@ZrP represent a combination of the individual spectra of α-ZrP and MTX, indicative of successful intercalation of MTX with no major chemical modification of either MTX or ZrP. As the molar ratio of MTX increases relative to ZrP, the following observations are made: 1) the relative intensity of the peaks corresponding to MTX increases; 2) the relative intensity of the α-ZrP peaks at 3590 cm^–1^ and 3510 cm^–1^ decreases, as expected due to water displacement by the intercalant upon intercalation;^71^ and 3) the α-ZrP shoulder at 1055 cm^–1^ is reduced in relative intensity, as expected since the P–O–H group proton acts as the exchangeable species during intercalation. These FTIR spectroscopy results, along with the XRPD results, confirm the successful intercalation of MTX into ZrP. As expected, the absolute value of the Z-potential for ZrP nanoparticles (−38.8 mV) is reduced upon MTX intercalation (−33.7 mV).

**Figure 3:**
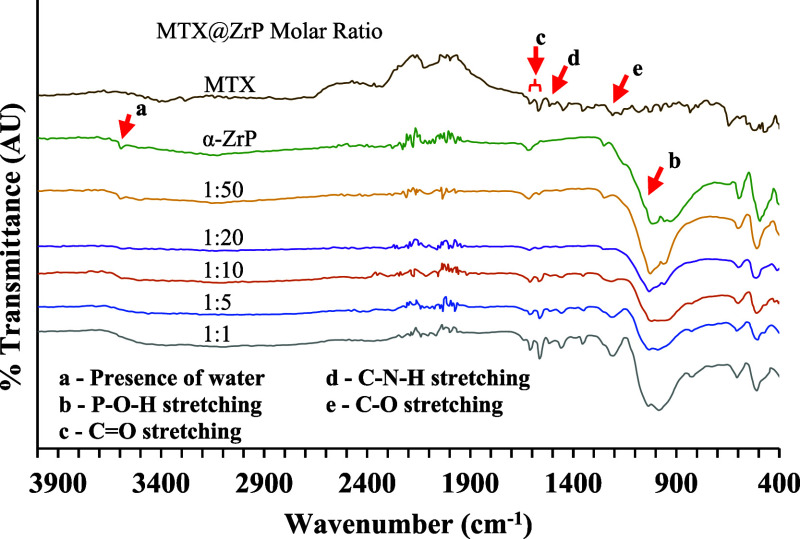
FTIR spectra of MTX, α-ZrP, and MTX@ZrP at different MTX:ZrP molar ratios.

Figure 4 shows the SEM image of MTX@ZrP. The SEM micrograph demonstrates that MTX@ZrP nanoparticles have a hexagonal platelet morphology, with an average size of 154 ± 18 nm, indicating that ZrP nanoparticles maintain their characteristic shape and morphology upon intercalation.^13^

**Figure 4:**
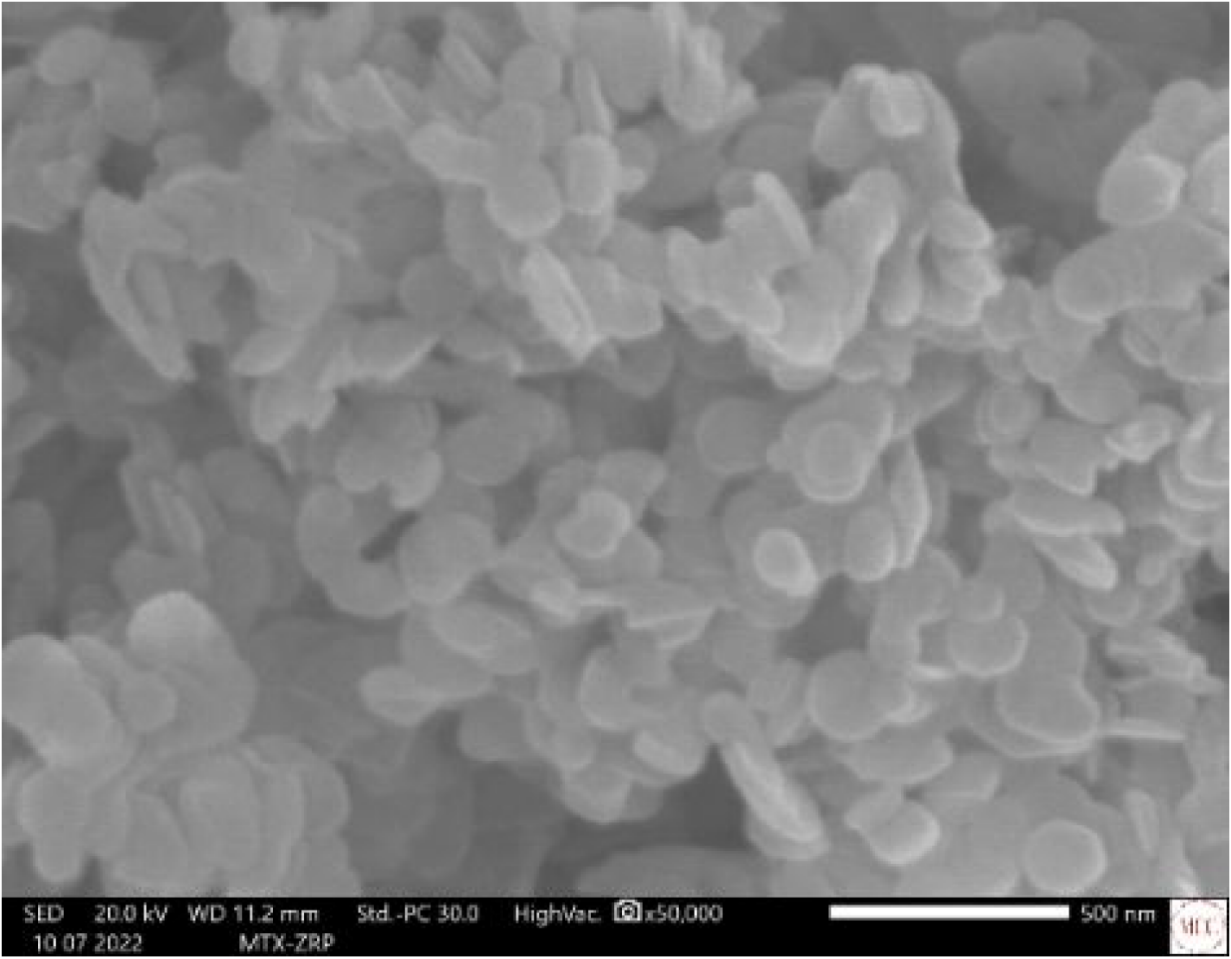
SEM image of MTX@ZrP. Scale bar: 500 nm.

TGA measurements were performed to quantify the amount of MTX intercalated into ZrP. Samples of each molar ratio of MTX@ZrP underwent a thermal decomposition process to monitor the percentage weight loss as a function of temperature. Figure 5a shows the TGA thermograms of ZrP and MTX@ZrP at different MTX:ZrP molar ratios. The thermograms show that the first significant weight loss occurs below 130 °C. This weight loss is due to the evaporation of water from MTX@ZrP. Given MTX’s hydrophilic nature, it is foreseeable that after vacuum drying, the material would absorb moisture from the environment when exposed, particularly on its surface and edges.^74^ This absorption increases the water content as the MTX-to-ZrP molar ratio increases.

**Figure 5:**
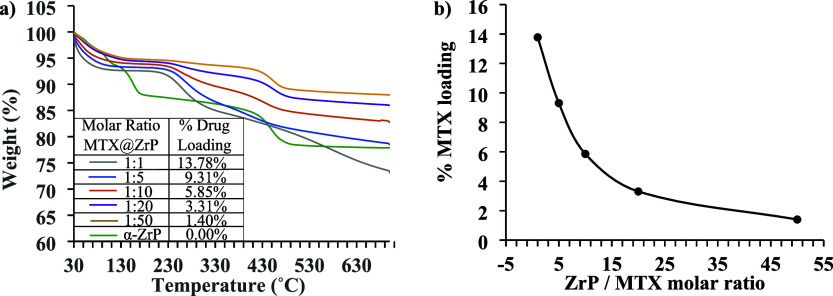
TGA thermograms of MTX@ZrP at different molar ratios. a) weight loss (%) as a function of temperature; b) MTX loading (%) as ZrP to MTX molar ratio.

Additionally, since MTX@ZrP was obtained as a mixed phase of ZrP, structural water molecules that remained in the interlaminar space and were not displaced during intercalation—responsible for the 3590 cm^–1^ band in the FTIR spectra^72^—also contribute to the mass loss seen in this temperature range.

The second substantial weight loss occurs between 230 °C and 490 °C, corresponding to the thermal decomposition of the intercalated MTX. Lastly, the final decomposition corresponds to the water loss resulting from the condensation of the phosphate groups in ZrP, forming zirconium pyrophosphate as the final thermal product.^75,76^ The results show that the 1:1 molar ratio of MTX@ZrP exhibited the highest intercalation loading at 13.8%, confirming the high drug-loading capacity of ZrP.^77^ Figure 5b shows that at a higher molar ratio of ZrP to MTX, there is lower MTX loading into ZrP.

In 2016, González et al.^21^ performed studies on the internalization mechanism of the anticancer drug doxorubicin (DOX) when it was intercalated into ZrP (DOX@ZrP) using MCF-7 human breast cancer and MCF-10A healthy breast cell lines. The results showed that the mechanism of cell internalization of ZrP nanoparticles is by clathrin-mediated endocytosis. Additionally, transmission electron microscopy revealed endosomes with ZrP nanoparticles encapsulated within them. Some of those endosomes were located near the plasma membrane, while others were located near the nucleus, suggesting that endocytic pathways transport ZrP nanoparticles rather than through passive diffusion. Therefore, given that MTX and DOX are planar polycyclic aromatic rings with similar structures, dimensions, and interlayer distances when intercalated in ZrP, the internalization of MTX@ZrP nanoparticles may also occur via endocytosis in tumor cells.^13,21^ In addition, Saxena et al. reported the cellular internalization of DOX@ZrP nanoparticles into MDA-MB-231 metastatic human breast cancer cells.^23^ These authors presented confocal laser scanning microscopy images that show enhanced cellular uptake of DOX@ZrP as compared to free DOX as well as localization of DOX@ZrP within the cell nucleus. Extensive literature on nanoparticle-based systems has established that cellular uptake generally occurs through endocytic mechanisms.^78–80^

Although further research is required to clarify the mechanism of MTX@ZrP cellular uptake, we envision that after endocytosis, nanoparticles will be enclosed in early endosomes, which mature and eventually fuse with lysosomes.^81,82^ This process implies that the initial pH of the cell surface, approximately 6.5 to 6.8, will gradually decrease to reach the lysosomal pH of 4.0–5.5.^82,83^ Therefore, to evaluate the stability and release of MTX from ZrP under physiological conditions, an in vitro DRT study was performed.

### 3.2 In Vitro DRTs

For this study, MTX@ZrP suspensions with the highest drug loading (1:1 MTX:ZrP molar ratio) were prepared individually in ALF and SBF. ALF, with pH = 4.5, mimics the lysosomal environment regarding ionic strength, composition, and ionic concentration, while SBF mimics the ionic concentration of human plasma, with pH = 7.4. MTX@ZrP suspensions with the highest drug loading (1:1 MTX:ZrP molar ratio) were individually prepared in ALF and SBF. During the filtration of each aliquot, an amount of the drug was retained on the filter. Consequently, it was necessary to correct the concentration values of each aliquot to account for the amount retained by the filter, as detailed in Section 2.5. Figure 6a presents the in vitro drug release profiles of MTX in ALF at pH 4.5, after concentration correction. The curve shows a release of 10.0% during the first 12 h of the study, which gradually increased to a cumulative release of 13.6% by day 8.

**Figure 6:**
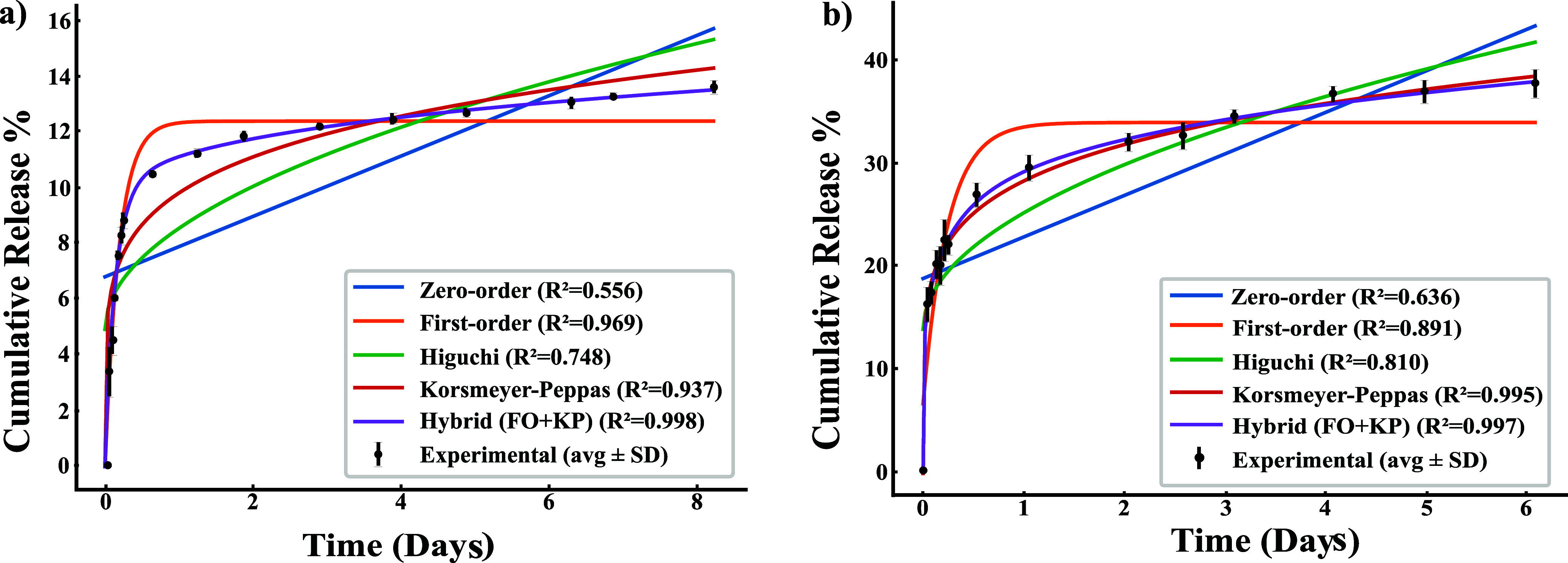
In vitro release profiles of MTX from MTX@ZrP (1:1 molar ratio) at 37 °C. a) ALF at pH = 4.5; b) SBF at pH = 7.4. Data are mean ± SD from three independent trials with model fits (zero-order, first-order, Higuchi, Korsmeyer–Peppas, and hybrid).

As shown in Figure 6b, the in vitro drug release profiles of MTX@ZrP were evaluated in simulated body fluid (SBF, pH 7.4) using classical kinetic models (zero-order, first-order, Higuchi, and Korsmeyer–Peppas). Model quality was assessed by the coefficient of determination (*R* ^2^).

In ALF, the release reached 10.0% during the first 12 h of the study, which gradually increased to a cumulative release of 13.6% by day 8. Zero-order (*R* ^2^ = 0.556) and Higuchi (*R* ^2^ = 0.748) models showed poor fits, suggesting that MTX release is not governed by constant-rate kinetics or simple diffusion. The first-order model provided the best fit (*R* ^2^ = 0.969), consistent with concentration-dependent release. The Korsmeyer–Peppas model also described the data well (*R* ^2^ = 0.937), with a fitted exponent *n* ≈ 0.18, pointing to anomalous, non-Fickian transport. A hybrid model, combining first-order and Korsmeyer–Peppas components, achieved the highest accuracy (*R* ^2^ = 0.998), supporting a mixed mechanism involving both concentration-driven kinetics and carrier-related structural effects.

In SBF, the release reached 37.6% by day 6, with an initial burst of 16.2% in the first hour. Zero-order and Higuchi models again performed poorly (*R* ^2^ = 0.636 and 0.810, respectively). The first-order model produced only a moderate fit (*R* ^2^ = 0.891), while the Korsmeyer–Peppas model provided an excellent description of the data (*R* ^2^ = 0.995), indicating predominant power-law behavior. The hybrid model further improved the fit (*R* ^2^ = 0.997), confirming that MTX release from ZrP in plasma-like conditions is best explained by a combination of mechanisms rather than a single kinetic process.

This behavior indicates that the release mechanism of MTX is pH-dependent, possibly due to changes in the drug’s protonation state and its interactions with ZrP. MTX is a weak base with two ionizable amino groups, with p*K*_a_ values of 5.99 for the -NH groups of the phenylenediamine and 8.13 for the amines in its side chains.^64,84^ Therefore, its net charge varies with pH: at pH = 7.4, MTX carries two positive charges, whereas at pH < 5.99, it becomes fully protonated, yielding four positive charges.^85^ During the intercalation of MTX, the suspension reaches a pH of ∼4.5, which is similar to that of lysosomes. Under these conditions, the four positive charges of MTX could be stabilized by hydrogen bonding or electrostatic interactions with the protonated phosphate groups of ZrP, as observed with DOX,^24,74^ thereby limiting its release.

In contrast, at pH 7.4, MTX shows reduced protonation, resulting in only two positive charges.^85^ This protonation reduction weakens the interaction strength between the drug and ZrP, facilitating the release of MTX from the ZrP, resulting in increased drug release. This difference in interaction strength between acidic and neutral conditions explains the observed pH-responsive release behavior.

Although SBF shows a 26.8% release of MTX during the first 12 h, an extended release is observed, reaching 37.6% by the sixth day. This extended release could benefit patients, as a MTX@ZrP could accumulate in the tumor tissue due to the EPR effect, and greater amount of MTX is released gradually through diffusion over time in the tumor microenvironment. With this extended release, the frequency of drug administration may be reduced, potentially decreasing acute toxicity. Ultimately, this approach could lessen the potential side effects and minimize damage to healthy tissues in patients receiving MTX treatment.^86,87^

### 3.3 In Vitro Cell Viability Assays

Figure 7 presents the results of MTX assay-based cell viability studies conducted on the androgen receptor-negative PC3 prostate cancer cell line. These studies evaluate the effects of ZrP, MTX@ZrP, and free MTX at equivalent MTX concentrations. The cell viability data indicate that after 24 h, the half-maximal inhibition concentration (IC_50_) for free MTX was 14.82 μM, corresponding to log­(IC_50_) = 1.17, whereas MTX@ZrP did not reach its IC_50_ value.

**Figure 7:**
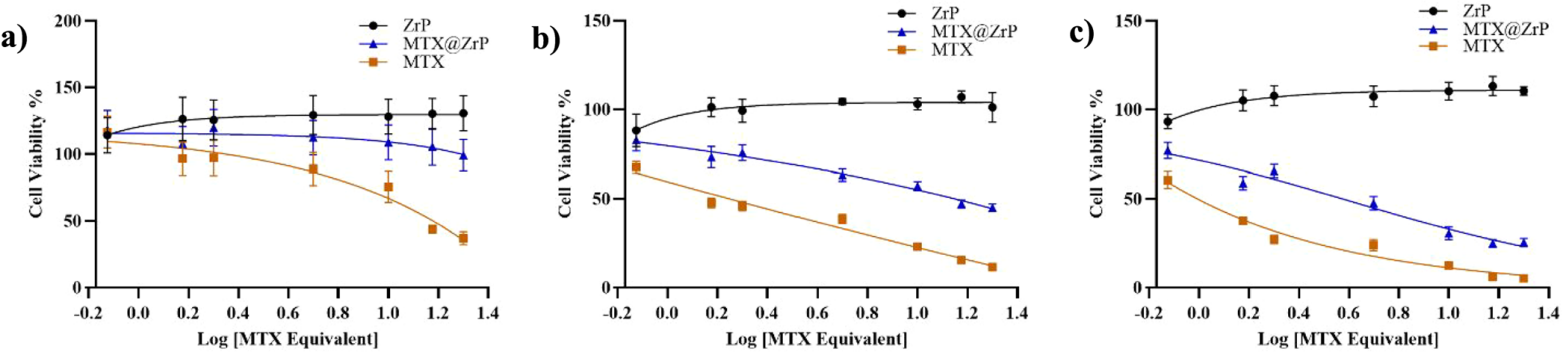
PC3 cell viability after a) 24 h, b) 48 h, and c) 72 h of exposure to ZrP, MTX@ZrP, and MTX at different MTX equivalents.

After 48 h, the IC_50_ for free MTX decreased to 1.74 μM, corresponding to log­(IC_50_) = 0.24, which is 8 times lower than the IC_50_ for MTX@ZrP, calculated at 13.94 μM or log­(IC_50_) = 1.14. After 72 h, the IC_50_ for free MTX was 0.98 μM, corresponding to log­(IC_50_) = −0.0076, which was 3.8 times lower than the IC_50_ for MTX@ZrP, obtained at 3.70 μM or log­(IC_50_) = 0.57.

Notably, the presence of ZrP in the equivalent MTX treatments did not induce cytotoxic effects in PC3 cells at 24, 48, or 72 h of exposure, which aligns with findings from previous studies.^21^ Interestingly, cell proliferation was higher at 24 and 48 h compared to the control cells. However, by 72 h, proliferation levels had become comparable to those of the control group.

These cell viability results suggest that, although MTX@ZrP is not as cytotoxic to PC3 prostate cancer cells as free MTX during the first 24 h of exposure, it exerts a more gradual cytotoxic effect over time. This sustained activity leads to a comparable reduction in cell viability at later time points, particularly at 72 h. The delayed yet effective response of MTX@ZrP supports its potential as a controlled-release formulation, capable of maintaining therapeutic efficacy while possibly reducing the acute toxicity associated with free MTX administration.

## 4 Conclusions

Structural and thermal characterization techniques (XRPD, FTIR, SEM, and TGA) confirmed the successful intercalation of MTX into ZrP, with dimensions suitable for exploiting the EPR effect in the passive accumulation of nanoparticles in tumor tissue. In vitro DRT demonstrated that 26.8% of MTX was released from ZrP under physiological conditions (pH 7.4, SBF buffer) during the first 12 h, reaching 37.6% by the sixth day. However, at an acidic pH (4.5, ALF buffer), which mimics the lysosomal environment, a gradual release of MTX was observed, reaching 13.8% by the eighth day. This pH-dependent and extended-release behavior at pH 7.4 is particularly advantageous, as it suggests that ZrP nanoparticles could reduce the immediate impact of MTX and the frequency of its administration. In addition, MTX@ZrP could also release the active drug in acidic environments, such as lysosomes, thus minimizing off-target toxicity.

Furthermore, cell viability assays with PC3 prostate cancer cells revealed that while free MTX showed rapid cytotoxicity, MTX@ZrP induces a gradual but sustained reduction in cell viability, achieving comparable effects at 72 h. This delayed response supports the potential use of ZrP as a controlled-release vehicle that can preserve therapeutic efficacy over time.
